# Aloperine Ameliorates IMQ-Induced Psoriasis by Attenuating Th17 Differentiation and Facilitating Their Conversion to Treg

**DOI:** 10.3389/fphar.2022.778755

**Published:** 2022-06-01

**Authors:** Hai-Feng Zhou, Fa-Xi Wang, Fei Sun, Xin Liu, Shan-Jie Rong, Jia-Hui Luo, Tian-Tian Yue, Jun Xiao, Chun-Liang Yang, Wan-Ying Lu, Xi Luo, Qing Zhou, He Zhu, Ping Yang, Fei Xiong, Qi-Lin Yu, Shu Zhang, Cong-Yi Wang

**Affiliations:** ^1^ NHC Key Laboratory of Respiratory Diseases, Department of Respiratory and Critical Care Medicine, The Center for Biomedical Research, Tongji Hospital, Tongji Medical College, Huazhong University of Sciences and Technology, Wuhan, China; ^2^ Department of Urology, Tongji Hospital, Tongji Medical College, Huazhong University of Sciences and Technology, Wuhan, China

**Keywords:** Psoriasis, Aloperine, Th17 differentiation, Th17 to Treg conversion, STAT3/STAT5 pathway

## Abstract

Aloperine is an anti-inflammatory compound isolated from the Chinese herb *Sophora alopecuroides L.* Previously, our group has reported that the generation of induced Treg was promoted by aloperine treatment in a mouse colitis model. However, the effect of aloperine on effector T cell subsets remains unclear. We therefore carefully examined the effect of aloperine on the differentiation of major subsets of T helper cells. Based on our results, psoriasis, a Th17 dominant skin disease, is selected to explore the potential therapeutic effect of aloperine *in vivo*. Herein, we demonstrated that topical application of aloperine suppressed epidermal proliferation, erythema, and infiltration of inflammatory cells in skin lesions. Mechanistic studies revealed that aloperine suppressed the differentiation of Th17 cells directly through inhibiting the phosphorylation of STAT3 or indirectly through impairing the secretion of Th17-promoting cytokines by dendritic cells. Moreover, aloperine enhanced the conversion of Th17 into Treg *via* altering the pSTAT3/pSTAT5 ratio. Collectively, our study supported that aloperine possesses the capacity to affect Th17 differentiation and modulates Th17/Treg balance, thereby alleviating imiquimod (IMQ)-induced psoriasis in mice.

## Introduction

CD4^+^ T helper cells play a pivotal role in the adaptive immune reaction associated with immune defense, immune surveillance, and immune homeostasis. These various functions are achieved through the differentiation of a variety of effector subsets, including Th1, Th2, Th17, follicular helper T cells (Tfh), and regulatory T cells (Treg) (Zhu and Paul, 2008). Upon interaction with the cognate antigen presented by antigen-presenting cells such as dendritic cells (DCs) in the periphery, CD4^+^ naïve T cells undergo a process of massive proliferation and differentiation into the distinct helper T cell subsets. The process of CD4^+^ T cell differentiation decision is governed predominantly by the cytokines in the microenvironment and the strength of the interaction of the T cell antigen receptor with the antigen. Many complex inflammatory diseases are caused by the disorder of these processes, such as type 1 diabetes, psoriasis, asthma, and so on (Sun et al., 2021; Yue et al., 2021). Although many therapies including glucocorticoids and immunomodulators are used in clinics, the development of novel, safe, and effective agents that target CD4^+^ T cells for treatment is further needed.

Aloperine is a kind of quinolizidine alkaloid extracted from *Sophora alopecuroides L.*, which has been used effectively in the treatment of various clinical disorders, such as dysentery, eczema, furuncle, and other skin inflammatory diseases (Wang et al., 2020). Recent studies including our own revealed that aloperine possesses the bioactivities against cancer, viral infection, oxidative stress, cardiovascular and neurological diseases (Wang et al., 2015; Hu et al., 2016; Fu et al., 2017; Ling et al., 2018; Zhang et al., 2018; Zhao et al., 2018; Chang et al., 2019; Li et al., 2020). Accumulating evidence shows that the clinical effects of the natural plant alkaloid are related to the up- or down-regulation of immune responses (Feng et al., 2006; Furusawa and Wu, 2007). Previously, our group demonstrated that oral administration of aloperine protects the mice against DSS-induced colitis through promoting Treg differentiation and activation (Fu et al., 2017). Thus, the effect of aloperine on the CD4^+^ T cell differentiation pattern catches our attention.

Psoriasis is a common chronic inflammatory skin disease characterized by the keratinocyte abnormal proliferation and differentiation, dermal blood vessel hyperplasia, and massive inflammatory infiltration (Mak et al., 2009; Lowes et al., 2014). According to epidemiologic survey, the morbidity rate of psoriasis in Asia is below 0.5%, while it is estimated to affect about 2–4% of the population in Western countries (Parisi et al., 2013; Boehncke, 2015). Psoriasis not only is a skin disease but also causes systemic disorders, such as psoriatic arthritis, cardiovascular disease, metabolic syndrome, and so on, which together contribute to a reduced quality of life and represent a mental stress on individuals and considerable economic burden on families and the society (Takeshita et al., 2017). Although the exact etiology of psoriasis is not fully understood, extensive studies have demonstrated it as a multifactorial disease.

As an immune-mediated disease, both innate and adaptive immune cells are involved in the pathogenesis of psoriasis, including different T-cell types such as Th17 cells, Th1 cells and Treg, dermal dendritic cells, macrophages, neutrophils, and natural killer cells (Deng et al., 2016). Robust evidence accumulated in the past years has shown that the IL-23/Th17 axis is central to the psoriatic pathology (Lowes et al., 2013; Girolomoni et al., 2017; Puig, 2017; Conrad and Gilliet, 2018). It is believed that trauma and infection can activate skin-resident dendritic cells and macrophages to produce IL-6, IL-23, and IL-12, which stimulate the maturation of Th17 and Th1 cells, respectively. IL-23 from inflammatory DCs has a great role in the amplification, activation, and pathogenic conversion of Th17 cells (Schirmer et al., 2010). Subsequent secretions of inflammatory cytokines, such as IL-17, IL-22, TNF-α, IFN-γ, and vascular endothelial growth factor (VEGF), stimulate keratinocyte proliferation and angiogenesis, which have important roles both in disease development and progression. On one hand, IL-17A stimulates keratinocyte proliferation and endothelial expression of P-selectins, E-selectins, and integrin ligands, including ICAM-1 and VCAM-1, to enhance neutrophil mobilization. On the other hand, activated keratinocytes produce large amounts of chemokines and antimicrobial peptides, which would in turn promote Th17 cell recruitment and produce more IL-17, resulting in a positive feedback loop that perpetuates the inflammatory response of psoriasis.

Currently, there is no cure for the spectrum of psoriatic diseases. By targeting the cytokine and immune networks in psoriasis, researchers have developed various therapeutic options, including topical agents (such as corticosteroids) and systemic immunosuppressive agents (such as methotrexate and cyclosporine). However, those current medications may have severe side effects, especially the high risk of infection. Thus, better and safer drugs and therapeutic strategies need to be developed. Previously, our group identified the role of aloperine in Treg induction and colitis treatment. In this study, we further revealed that the administration of aloperine modulates Th17/Treg balance by targeting the STAT3/STAT5 signaling pathway and/or indirectly through affecting the recruitment and activation of antigen-presenting cells in psoriatic skin lesions, which render it to be a potential candidate drug to psoriasis in clinical settings.

## Materials and Methods

### Animals

Healthy male C57BL/6 mice (8–10 weeks old) were purchased from Beijing Vital River Laboratory Animal Technology Co., Ltd. (Beijing, China). The mice were bred at the Tongji Medical College and maintained under SPF conditions for at least 1 week before any experiment. All animal studies were approved by the Animal Care and Use Committee in Tongji Hospital (TJH-201612001).

### Psoriasis Model and Aloperine Treatment

Aloperine (Shanghai Yuan-ye Bio-Technology Co., Ltd.) was made of 2% cream by mixing in the emollient cream vehicle. Imiquimod (IMQ) cream was purchased from Sichuan Mingxin Pharmaceutical. Twenty mice were randomly divided into the treatment group (2% aloperine + imiquimod) and vehicle control group (vehicle + imiquimod). The mice were shaved on the back, and then subjected to 5% Aldara (a brand of IMQ) at a dose of 62.5 mg daily on the back skin for 5 consecutive days, and aloperine or vehicle cream was administered at the same time or after the psoriasis model was established.

### Scoring of Psoriatic Skin Inflammation

The severity of inflammation of the back skin was evaluated by an objective scoring system, which was based on the Psoriasis Area and Severity Index (PASI). Erythema, scaling, and anabrosis were scored independently from 0 to 4 as follows: 0, none; 1, slight; 2, moderate; 3, marked; and 4, very marked. The cumulative score (erythema plus scaling plus thickening) was served to indicate the severity of inflammation (scale 0–12).

### Histological Analysis

The skin tissue samples were collected and fixed in 4% paraformaldehyde for 24 h at room temperature and embedded in paraffin. The 4-µm sections were subjected to hematoxylin and eosin (H&E) staining as previously described. Similarly, partial sections were stained with the Ki-67 antibody, and developed by 3,3'-diaminobenzidine as reported (Yue et al., 2021).

### Mouse Naïve CD4^+^ T-Cell Isolation and Differentiation

CD4^+^CD44^low^CD62L^high^ naïve T-cells were extracted from spleens and lymph nodes of 10-week-old C57BL/6 male mice with a mouse naïve CD4^+^ T-cell isolation kit (catalog number 19782, STEMCELL Technologies EasySep^™^, CA, United States), and the purity of isolated cells was >90%. The isolated naïve CD4^+^ T-cells were seeded into culture wells coated with 10 μg/ml anti-CD3 and 10 μg/ml anti-CD28 in sterile phosphate-buffered saline (PBS) and incubated overnight at 4°C. Furthermore, different subsets were differentiated by Th1, Th2, and Th17 polarizing conditions with or without aloperine (10 nM). For the Th1 cells, naïve T-cells were incubated with 10 ng/ml IL-2 (eBioscience, San Diego, CA, United States), 10 ng/ml IL-12 (eBioscience, San Diego, CA, United States), and 10 µg/ml anti-IL-4. For the Th2 cells, naïve T-cells were incubated with 10 ng/ml IL-4 (#214–14) and 10 µg/ml anti-IFN-γ. For the Th17 cells, naïve T-cells were incubated with 10 ng/ml IL-1β, 10 ng/ml IL-6, 10 ng/ml IL-23, 2.5 ng/ml TGF-β, 10 µg/ml anti-IL-4, and 10 µg/ml anti-IFN-γ. For the Th17 cell conversion experiment, Th17 cells were differentiated as described above, and, the medium was then replaced with the Treg differentiation cytokine cocktail after washing three times on day 3 and incubated for 2 days. The cells were next subjected to flow cytometry analysis.

### Flow Cytometry Analysis

Single-cell suspensions were prepared from the spleens, skin tissues, and exudates or recovered from cell cultures. The isolated skin tissues were digested with collagenase D (Roche Applied Science) solution (400 U/ml) for 4–6 h at 37°C with periodic agitation. Ethylenediaminetetraacetic acid (10 mM final concentration) was added to the collagenase-digested cells for 1 min and then quenched with cold PBS. The cells were stained with the indicated fluorescently labeled antibodies at 4°C in the dark for 30 min and then washed with fluorescence advanced cell sorting buffer (2% bovine serum albumin in PBS). Detection of intracellular molecules was performed; the cells were permeabilized and stained intracellularly as previously described (Zhong et al., 2014). Flow cytometric measurements were performed with an LSR Fortessa flow cytometer (BD Biosciences, Franklin Lakes, NJ, United States), and FlowJo software was used for the subsequent data analysis as instructed. The PerCP-conjugated antimouse CD45 (#103130), FITC-conjugated antimouse CD4 (#100406), PE-conjugated antimouse CD44 (#103036), APC-conjugated antimouse CD62L (#104412), PE-conjugated antimouse CD11b (#101207), APC-conjugated antimouse CD11C (#117310), PE-conjugated antimouse LY6G (#127607), APC/Cy7-conjugated antimouse Ly6C (#128026), FITC-conjugated antimouse F4/80 (#123108), PE/Cy7-conjugated antimouse IFN-γ (#505826), APC-conjugated antimouse IL-4 (#504106), APC-conjugated antimouse IL-17A (#506916), PE-conjugated antimouse IL-17 (#506904), and Alexa Fluor^®^647-conjugated antimouse Foxp3 (#126408) antibodies were purchased from BioLegend (San Diego, CA, United States).

### Western-Blot Analysis

Western-blot assays were performed as described previously (He et al., 2018). Briefly, the prepared cell samples were lysed and then separated on 10% (vol/vol) polyacrylamide gels and transferred onto PVDF membranes. The membrane was probed with antibodies (1: 1000 dilution) including P-STAT3, RORγt, P-STAT5 (Cell Signaling Technology, Danvers, MA, United States), and β-actin (Santa Cruz Biotechnology, Santa Cruz, CA, United States), followed by probing to the corresponding horseradish peroxidase-conjugated secondary antibody, respectively. The reactive bands were developed using the established techniques (Yao et al., 2016). The intensity of each band was analyzed using the densitometry feature in the ImageJ software.

### Quantitative RT-PCR Analysis

The part of injured skin tissues and spleens or culture cells were collected and subjected to RNA isolation using the Trizol^™^ reagent (Takara, Japan) as instructed. For the syntheses of complementary DNA, an aliquot containing 1 μg of total RNA was reverse-transcribed using a cDNA synthesis kit (Takara, Japan). Real-time polymerase chain reaction (PCR) was performed using the SYBR Green PCR master mix (Applied Biosystems, South San Francisco, CA, United States) in an ABI Prism 7500 Sequence Detection System (Applied Biosystems, South San Francisco, United States). The β-actin gene was used as a reference to normalize the data, and the PCR primers used for PCR amplification are listed in Table 1. The relative quantitative levels for each of target genes were analyzed using the 2^−ΔΔCT^ method as previously reported (Fu et al., 2017).

**TABLE 1:** Primers used for the real-time PCR.

Target gene	Forward sequence (5′-3′)	Reverse sequence (5′-3′)
m-IL-6	5′-TAC​CAC​TTC​ACA​AGT​CGG​AGG​C-3′	5′-CTG​CAA​GTG​CAT​CAT​CGT​TGT​TC-3′
m-IL-17A	5′-CAG​ACT​ACC​TCA​ACC​GTT​CCA​C-3′	5′-TCC​AGC​TTT​CCC​TCC​GCA​TTG​A-3′
m-IL-23	5′-CAT​GCT​AGC​CTG​GAA​CGC​ACA​T-3′	5′-ACT​GGC​TGT​TGT​CCT​TGA​GTC​C-3′
m-IL-1β	5′-TGG​ACC​TTC​CAG​GAT​GAG​GAC​A-3′	5′-GTT​CAT​CTC​GGA​GCC​TGT​AGT​G-3′
m-RORγt	5′-GTG​GAG​TTT​GCC​AAG​CGG​CTT​T-3′	5′-CCT​GCA​CAT​TCT​GAC​TAG​GAC​G-3′
m-IL-10	5′-CGG​GAA​GAC​AAT​AAC​TGC​ACC​C-3′	5′-CGG​TTA​GCA​GTA​TGT​TGT​CCA​GC-3′
m-Foxp3	5′-CCT​GGT​TGT​GAG​AAG​GTC​TTC​G-3′	5′-TGC​TCC​AGA​GAC​TGC​ACC​ACT​T-3′
m-TGF-β	5′-TGA​TAC​GCC​TGA​GTG​GCT​GTC​T-3′	5′-CAC​AAG​AGC​AGT​GAG​CGC​TGA​A-3′
m-IFN-γ	5′-CAG​CAA​CAG​CAA​GGC​GAA​AAA​GG-3′	5′-TTT​CCG​CTT​CCT​GAG​GCT​GGA​T-3′
m-TNF-α	5′-GGT​GCC​TAT​GTC​TCA​GCC​TCT​T-3′	5′-GCC​ATA​GAA​CTG​ATG​AGA​GGG​AG-3′
m-β-actin	5′-CAT​TGC​TGA​CAG​GAT​GCA​GAA​GG-3′	5′-TGC​TGG​AAG​GTG​GAC​AGT​GAG​G-3′

### Air Pouch Model

The air pouch model was created according to the previous studies (Gaspar et al., 2014). Ten mice, whose back sides were cautiously shaved to clean the area, were randomly allocated into two groups: the LPS + PBS group and LPS + aloperine group. Dermal air pouches were generated by injecting the mice on dorsal sites with 3 ml of filtered air (0.20 µm filter) on day 0, and another 3 ml of sterile air was injected on day 3 for re-expansion. On day 6, 1 ml of sterile saline solution containing 10 ng/ml LPS (Sigma, St. Louis, United States) with or without aloperine (4 mg/ml) was injected into the preformed air pouches in mice from the LPS + PBS group and LPS + aloperine group, respectively. Twelve hours later, the exudates of the pouches were collected after injection of 1 ml of saline solution containing 20 U/ml heparin and 2% fetal calf serum (FCS), followed by 1 min of gentle massage. This procedure was repeated twice, and the total collections were washed with cold PBS and centrifuged (5 min, 300 g), followed by suspending with PBS. The number of cells in the pouch fluid samples was determined using a hemocytometer. In addition, different cell types of inflammatory exudates were subjected to flow cytometry analysis as above.

### Activation of BMDC and Supernatant Transfer

Bone marrow-derived dendritic cells (BMDCs) were generated from C57BL/6 mice (8–10 weeks old) as previously described (Zhong et al., 2010). Briefly, the mice were sacrificed, and the bone marrow cells were flushed out from the femurs and tibias and then treated with ACK lysis buffer. Pooled cells were suspended to 2 × 10^6^/ml and cultured in RPMI 1640 supplemented with 10% fetal bovine serum, 100 U/ml penicillin, 100 g/ml streptomycin, 50 μM mercaptoethanol, 10 mM *N*-(2-hydroxyethyl)piperazine-*N’*-ethanesulfonic acid, recombinant murine granulocyte macrophage colony stimulating factor (GM-CSF) (10 ng/ml), and interleukin (IL)-4 (10 ng/ml) (#214–14) at 37°C in 5% CO_2_ atmosphere. The nonadherent cells were discarded after 48 h of culture, and the adherent cells were cultured with a fresh medium containing rmGM-CSF and IL-4 on alternative days. On day 7, 100 ng/ml LPS (Sigma, St. Louis, United States) was added for 12 h to induce the activation of BMDCs with or without pretreatment of aloperine (10 nM) for 1 h. The cells were next subjected to analysis of RT-PCR, western blotting, and flow cytometry. For supernatant transfer, the LPS + PBS- or LPS + aloperine-treated BMDCs were washed and cultured in the fresh complete medium for another 24 h. Then, the supernatants were collected to culture the CD4^+^ T-cells in the presence of TCR stimulation. The CD4^+^ T-cells were harvested for the RT-PCR analysis 48 h later.

### Statistical Analysis

All *in vitro* experiments were based on at least three independent biological replications.

An independent Student’s t-test was used to examine the significant differences between the two groups. Statistical analysis of the data was performed using the GraphPad Prism 5 software (GraphPad Software Inc., San Diego, CA, United States), and the results were expressed as the mean ± standard error of the mean (SEM). For all statistics, a *p* value <0.05 was regarded as statistically significant.

## Results

### Topical Application of Aloperine Ameliorates Psoriasis Induced by IMQ

**FIGURE 1:**
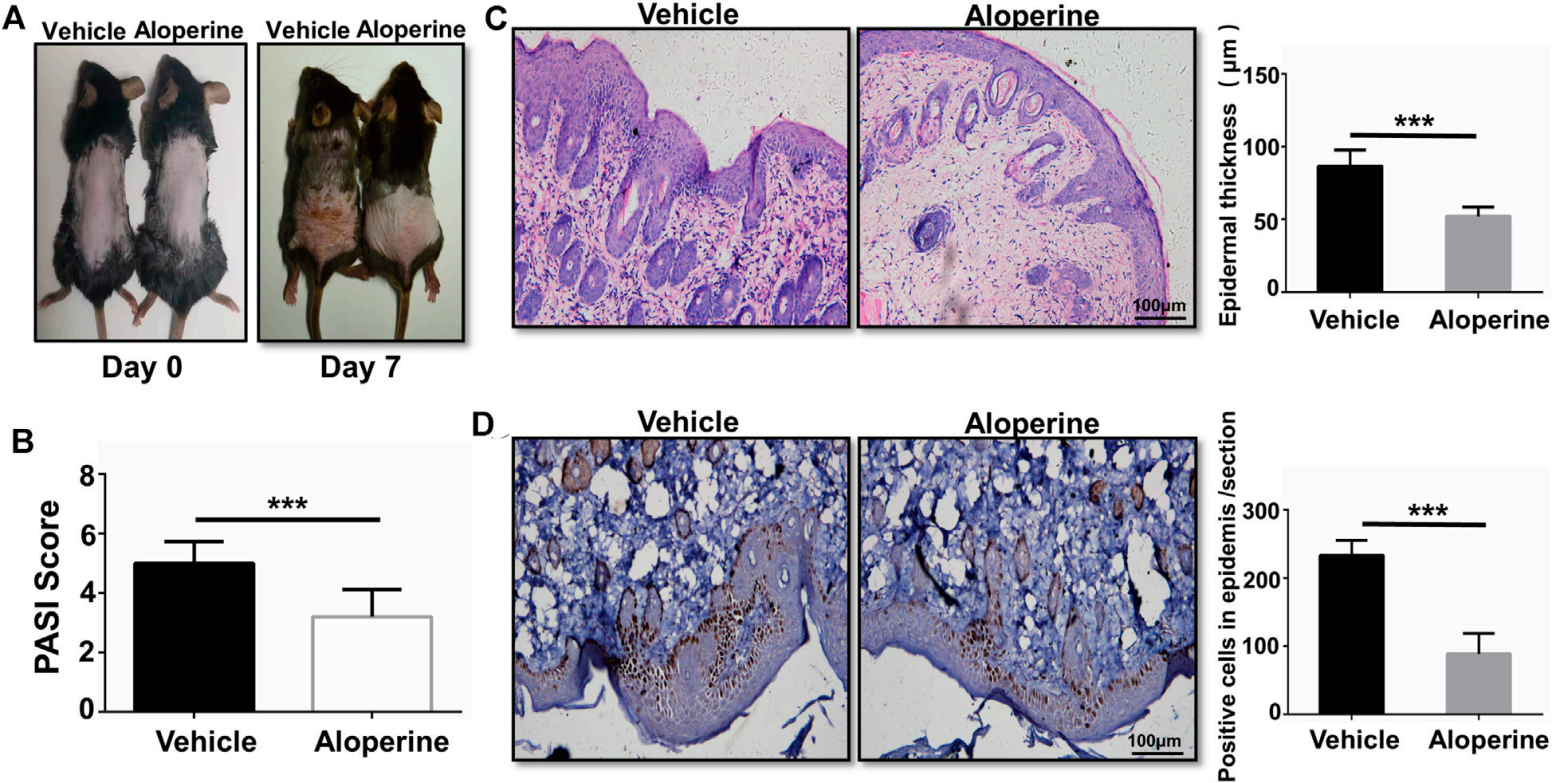
Topical application of aloperine ameliorates psoriasis induced by IMQ. **(A)** Representative presentation of the back skin at day 7 of the IMQ treatment. **(B)** The severity was graded by a modified PASI. **(C)** Histological analysis of the mouse back skin by H&E staining. **(D)** Histological analysis of IHC staining for Ki-67. Corresponding quantification analyses of epidermal thickness and positive proliferation cells were carried out as described. The values are presented as the mean ± SEM (*n* = 10 mice per group). ∗*p* < 0.05; ∗∗*p* < 0.01; and ∗∗∗*p* < 0.001.

First of all, we examined the therapeutic effect of aloperine on psoriasis, a skin lesion predominantly mediated by the CD4^+^ T-cells. To this end, we applied IMQ on the shaved back skin of B6 mice with or without 2% aloperine cream once per day for 5 consecutive days (Chuang et al., 2018). All the mice in the control group manifested significant general skin symptoms, including erythema, silver plaques, and anabrosis after 7 days (Figure 1A). Remarkably, topical application of 2% aloperine cream significantly ameliorated pathological situations of skin lesions when compared with the control group (Figure 1A). In addition, as a marker of severity in psoriasis, the mice treated with aloperine exhibited lower average PASI scores than the control group (Figure 1B).

To further confirm the above data, histological analysis was next conducted. In comparison to the control group, H&E staining of the skin lesion originating from the aloperine treatment group manifested a significantly lower thickness of the epidermis layer along with the attenuated IMQ-induced psoriasis (Figure 1C). In terms of Ki-67 immunohistochemistry staining, fewer positive cells with brown particles were observed in the basal layer cells of the lesion skin in the aloperine groups (Figure 1D). Additionally, we tested the effect of aloperine on the already established psoriasis to determine whether it has potential to serve as a therapeutic agent. The mouse model of psoriatic dermatitis was first induced by IMQ. Then, the mice received aloperine or control cream (Supplementary Figure S1A). The results showed that aloperine significantly alleviated the skin damage compared to control treatment (Supplementary Figures S1B–D). Furthermore, aloperine suppressed the local immune response (Supplementary Figures S2A,B), which was consistent with the results presented above. Altogether, these results suggested that aloperine treatment ameliorated IMQ-induced psoriatic skin injury in mice.

### Aloperine Inhibits the Activation of DCs

**FIGURE 2:**
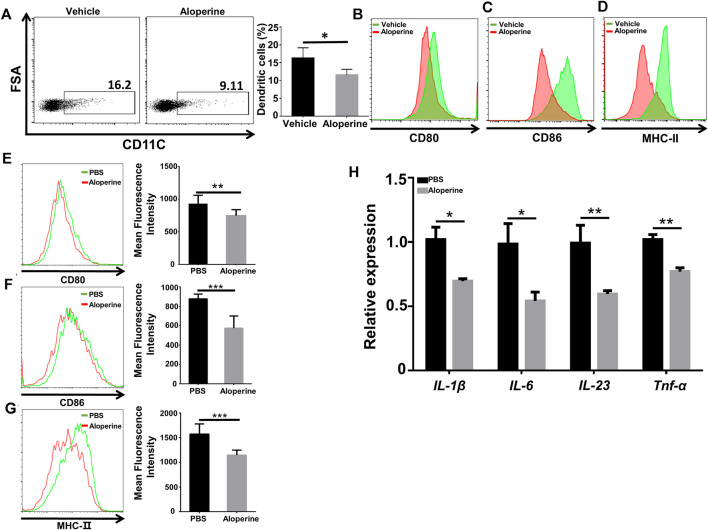
Aloperine inhibits the activation of DCs. **(A)** The proportion of CD11C^+^ DCs among CD45^+^ cells in the skin lesion. **(B–D)** Mean Fluorescence Intensity (MFI) analysis of the expression of CD80, CD86, and MHC-Ⅱ on skin DCs. **(E–G)** MFI analysis of the expression of CD80, CD86, and MHC-Ⅱ in *in vitro* differentiated BMDCs. **(H)** Real-time PCR analysis of cytokine expression (IL-1β, IL-6, IL-23, and TNF-α) in DCs. The data are shown as the mean ± SEM from three independent experiments. ∗*p* < 0.05; ∗∗*p* < 0.01; and ∗∗∗*p* < 0.001.

Previous studies have reported that specialized APC subsets of the dermal dendritic cells are important in the initiation and development of psoriatic skin inflammation (Xiao et al., 2017; Wang and Bai, 2020). Also, aloperine has been recognized as a potent anti-inflammatory drug. We thus tested whether aloperine could affect the activation of the dendritic cells, thereby reducing cytokine secretion, especially IL-6 and IL-23, to reduce Th17 differentiation and proliferation indirectly for the improved skin local inflammatory environment. In view of this, we first examined the frequency and activation state of DCs among CD45^+^ cells in the skin lesion. We observed a decreased DC population along with reduced activation markers (CD80, CD86, MHC-Ⅱ) in the aloperine group (Figures 2A–D). In line with the *in vivo* data, we then checked the effect of aloperine on the activation of DCs *in vitro*. As shown in Figures 2E–G, the expressions of CD80, CD86, and MHC-Ⅱ were significantly decreased upon aloperine treatment. Moreover, aloperine reduced the expression of IL-1β, IL-6, IL-23, and TNF-α in BMDCs (Figure 2H). These findings suggest that aloperine plays a crucial role in preventing the activation of skin DCs, which leads to a microenvironment disfavoring Th17 development.

### Aloperine Inhibits the Migration and Th17-Inducing Effect of DCs

**FIGURE 3:**
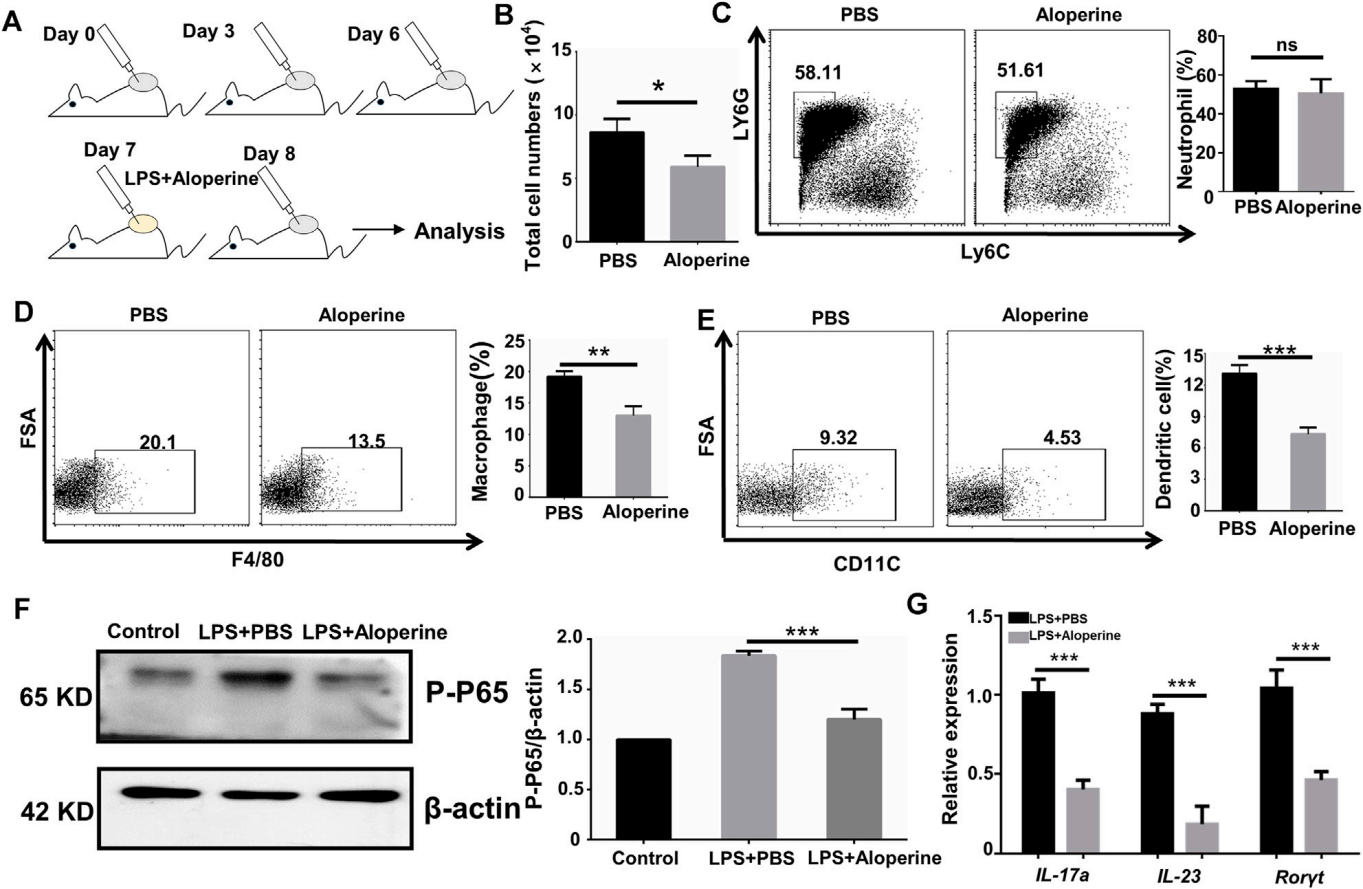
Aloperine inhibits the migration and Th17-inducing effect of DCs. **(A)** A graphical presentation of the air pouch model. On the first day, 3 ml of sterile air was injected subcutaneously into a shaved skin site on the back of each mice. Then, the pouches were settled for 3 days to heal the wound. On the third day, this action was repeated. On the sixth day, LPS was injected into the pouches with or without aloperine. The exudates were collected for subsequent analysis 24 h later. **(B)** Quantitative analysis of total cell numbers in the skin. **(C–E)** Representative flow cytometry analysis of neutrophils (CD11b^+^MHC-II^−^Ly6G^+^Ly6C^−^), macrophages (CD11b^+^F4/80^+^), and DCs (CD11c^+^F4/80^−^). **(F)** Western-blot analysis for p-P65 and β-actin. **(G)** Real-time PCR analysis of IL-17A, IL-23, and RORγt in CD4^+^ T-cells receiving DC cultural supernatant pretreated by LPS + PBS or LPS + aloperine. The values are presented as the mean ± SEM (*n* = 5 mice per group). ∗*p* < 0.05; ∗∗*p* < 0.01; and ∗∗∗*p* < 0.001.

An important characteristic for psoriasis is the accumulation of myeloid cells, including neutrophils, macrophages, and DCs (Schön et al., 2017; Han et al., 2020; McGinley et al., 2020). Thus, we were interested in whether aloperine would have an effect on the migration of myeloid cells. An air pouch inflammatory model in mice is considered a useful and convenient experiment *in vivo* for determining the anti-inflammatory activity of the testing compounds (Vandooren et al., 2013; Gaspar et al., 2014). A general outline of the method used is shown in Figure 3A. As expected, aloperine decreased the total number of inflammatory cells in the exudate induced by LPS (Figure 3B). Surprisingly, the neutrophil infiltration was comparable between the groups, suggesting that the decreased neutrophil cellularity in psoriatic lesions might be a result of aloperine-mediated Th17 cell inhibition (Figure 3C). In contrast, the trafficking of antigen-presenting cells, DCs in particular, was significantly decreased in the aloperine treatment group (Figures 3D,E).

Furthermore, we demonstrated a decreased phosphorylation level of P65 after aloperine treatment (Figure 3F), implying that aloperine could inhibit the NF-κB signaling pathway. By supernatant transfer and subsequent RT-PCR analysis, we showed that mRNA levels for the Th17-associated molecules including IL-17A, IL-23, and RORγt were decreased in the CD4^+^ T-cells receiving the supernatant of DCs pretreated by aloperine (Figure 3G). Altogether, these findings support that aloperine could suppress local inflammation in psoriatic skin lesions through inhibiting the migration, activation, and Th17-inducing capacity of the DCs.

### Aloperine Decreases the Th17/Treg Ratio in the Psoriasis-like Lesions

**FIGURE 4:**
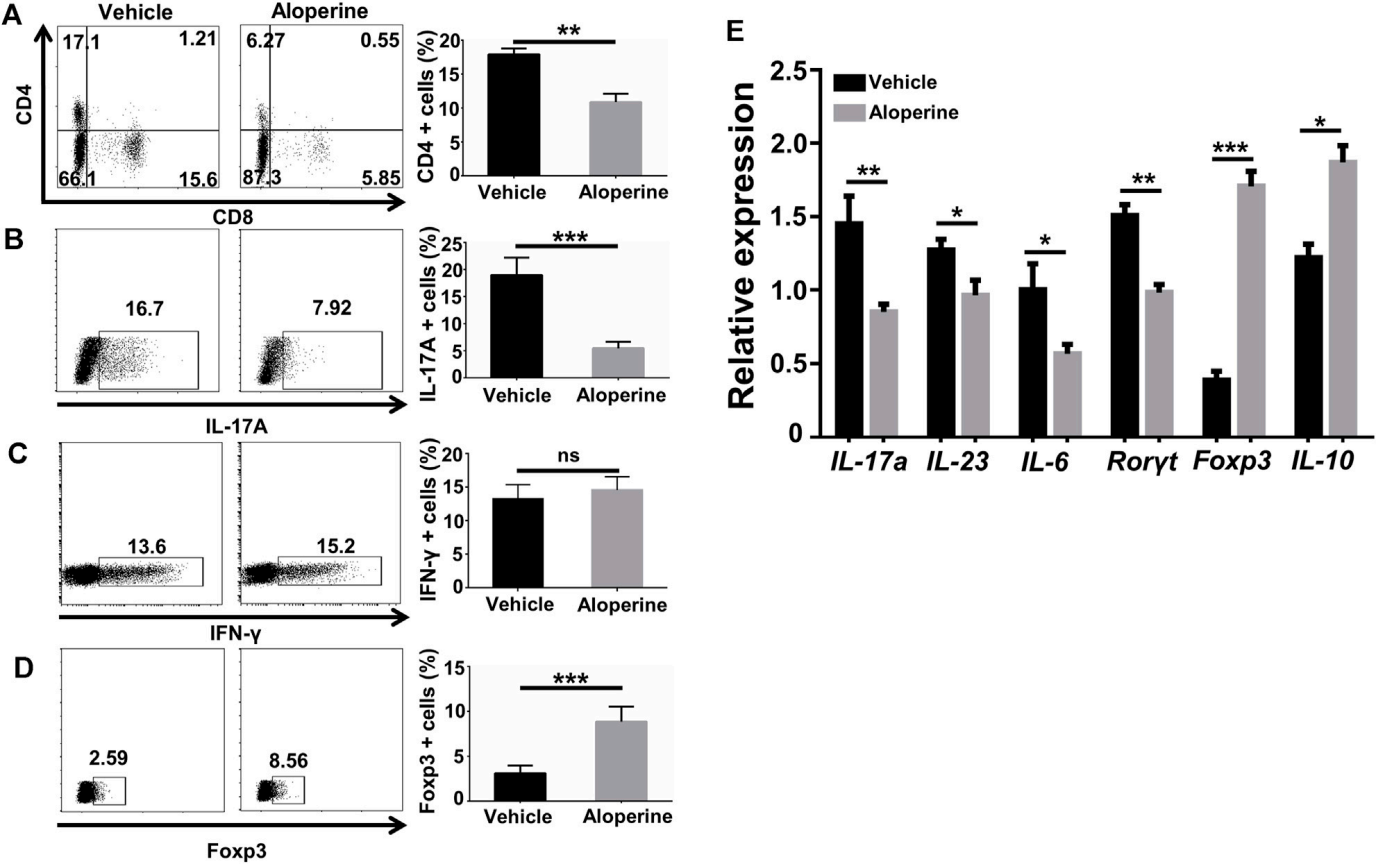
Aloperine decreases the Th17/Treg ratio in the psoriasis-like lesions. **(A–D)** Flow cytometry analysis for CD4, CD8, Th1, Th17, and Treg cells in the skin lesions. **(E)** Real-time PCR analysis of IL-17A, IL-23, IL-6, RORγt, Foxp3, and IL-10. The values are presented as the mean ± SEM (*n* = 8 mice per group). ∗*p* < 0.05; ∗∗*p* < 0.01; and ∗∗∗*p* < 0.001.

To understand the effect of aloperine on the CD4^+^ T-cells, a single-tissue cell suspension was made from the skin lesion by the digestion of collagenase and hyaluronidase for flow cytometry analysis when the mice were sacrificed. In comparison to the vehicle control group, aloperine significantly decreased the infiltration of CD4^+^ and CD8^+^ T-cells (Figure 4A), indicating a reduced adaptive immune response. Furthermore, the frequencies of IL-17^+^ Th17 cells were substantially decreased in the aloperine treatment group (Figure 4B), while IFN-γ^+^ Th1 cells displayed no perceptible difference (Figure 4C). On the contrary, as shown in (Figure 4D), the Treg cells were significantly increased in the aloperine treatment group.

RT-PCR was next performed to determine the mRNA levels of cytokines and key transcription factors for Th17 and Treg in the skin lesions. Consistent with the flow cytometry results, the levels of IL-17A, IL-23, IL-6, IFN-γ, and RORγt mRNA in the skin lesions were significantly lower than that in vehicle control mice. In contrast, aloperine markedly increased IL-10 and Foxp3 mRNA levels, the characteristic of immunomodulatory Tregs (Figure 4E). Taken together, aloperine treatment decreased the Th17/Treg ratio in skin lesions, thereby alleviating immune-mediated pathology in the psoriasis induced by IMQ.

### Aloperine Directly Affects Th17 *In Vitro* Differentiation *Via* Inhibiting the STAT3 Signalling

**FIGURE 5:**
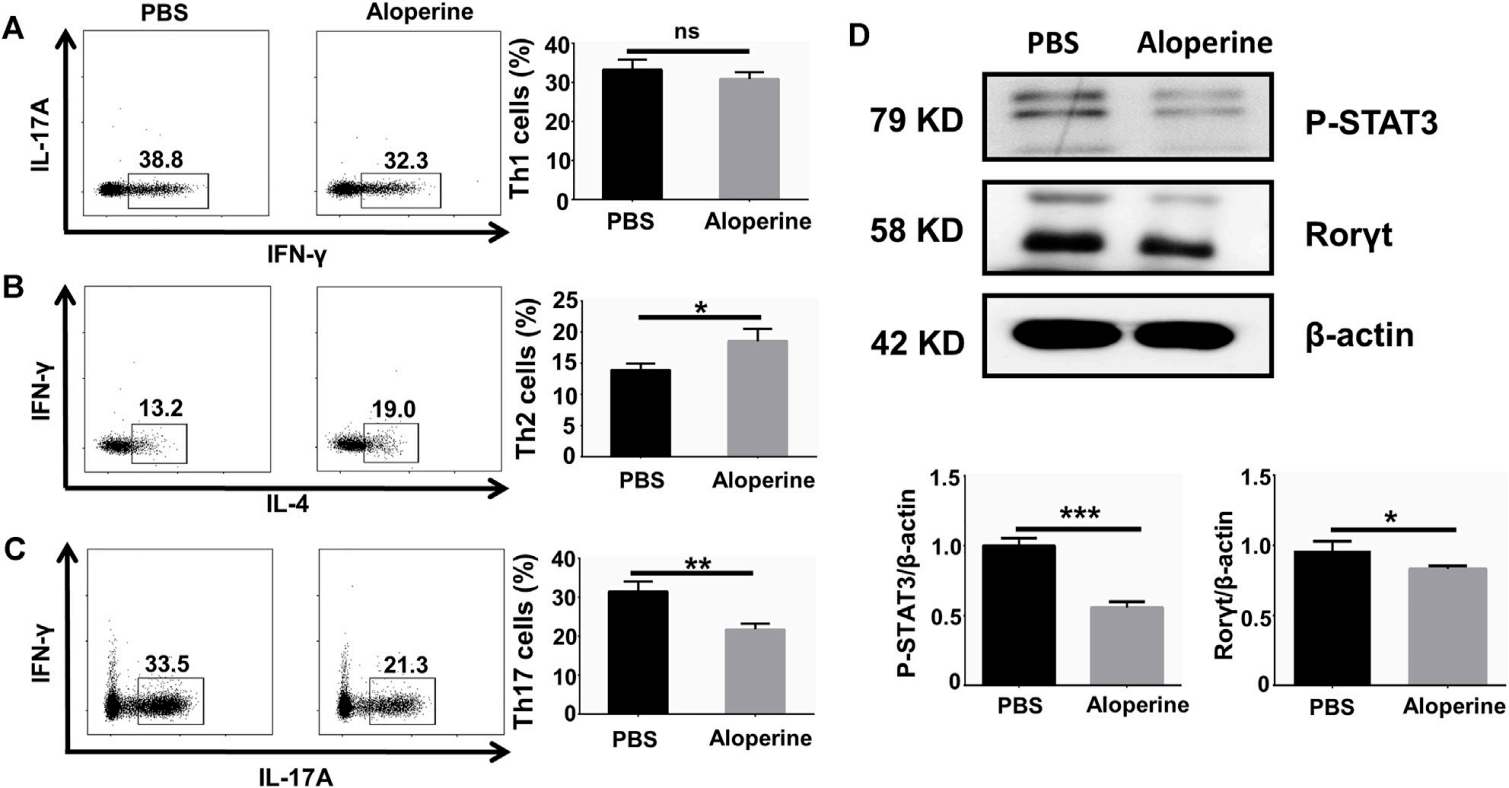
Aloperine directly affects Th17 *in vitro* differentiation by inhibiting the STAT3 signalling. **(A–C)** IFN-γ^+^ Th1, IL-4^+^ Th2, and IL-17A^+^ Th17 polarizing efficiency were analyzed by flow cytometry. **(D)** The levels of RORγt and phosphorylated STAT3 were assessed by western blotting. The results were from three independent experiments. The values are presented as the mean ± SEM. ∗*p* < 0 05; ∗∗*p* < 0.01; ∗∗∗*p* < 0.001; and ns, *p* ≥ 0.05.

In our previous study, we confirmed that aloperine promoted Treg differentiation and activation through suppressing the PI3K/Akt/mTOR signaling pathway both *in vivo* and *in vitro* (Fu et al., 2017). This phenomenon raised our interest to further examine the effects of aloperine on the CD4^+^ T-cell differentiation. To address this issue, naïve CD4^+^ T-cells isolated from WT B6 mice were differentiated toward specific effector populations in the presence of cytokine cocktails and α-CD3/α-CD28 with or without aloperine as described, including Th1, Th2, and Th17 subsets. Apparently, our flow cytometry analysis showed that no change in CD4^+^ IFN-γ^+^ Th1 cell frequency was observed after aloperine treatment (Figure 5A). On the contrary, treatment of aloperine resulted in a moderate increase in the IL-4^+^ Th2 cells when compared with the control (Figure 5B). Interestingly, aloperine was able to promote a significant decrease in the CD4^+^ IL-17A^+^ Th17 cell frequencies (Figure 5C).

Accumulative evidence has shown that the JAK/STAT signaling pathways are essential for the differentiation of Th17 cells. In particular, the phosphorylation of STAT3 is important for the expression of RORγt, which is a key transcription factor in the process of Th17 differentiation, by TGF-β in combination with IL-6 and IL-23. To dissect the mechanism by which aloperine treatment impairs the Th17 program, we examined the phosphorylation of STAT3 by western blotting. Our results showed that aloperine markedly inhibited the phosphorylation of STAT3 and the expression of RORγt under the Th17-polarizing conditions (Figure 5D). Collectively, these results indicate that aloperine not only promotes Treg differentiation but also directly impedes the Th17 polarizing program through inhibiting the phosphorylation of STAT3.

### Aloperine Facilitates the Conversion of Th17 to Treg

**FIGURE 6:**
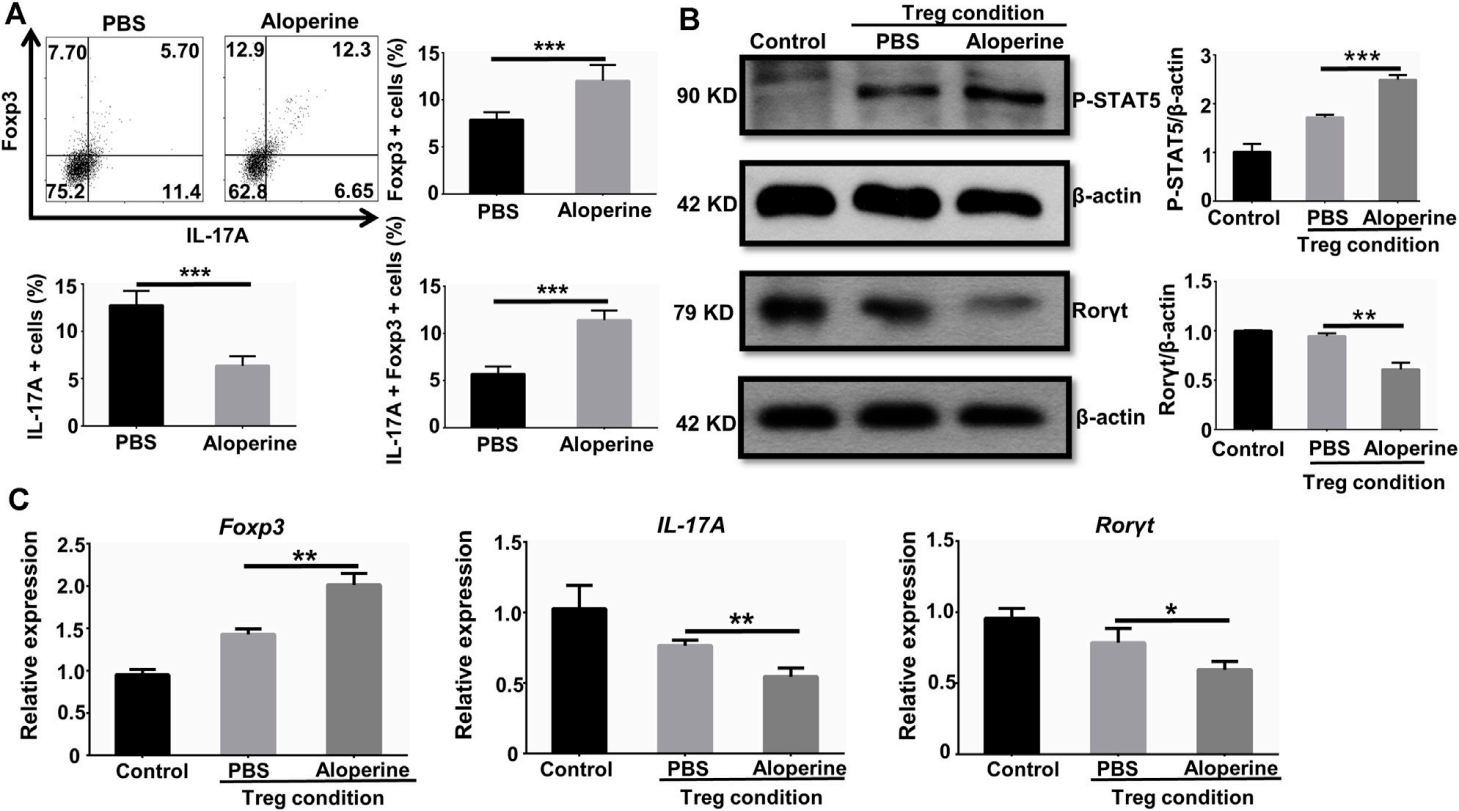
Aloperine facilitates the conversion of Th17 to Treg. **(A)** Flow cytometry analysis of the conversion of Th17 into Treg. **(B)** Western-blot analysis was conducted to assess the influence of aloperine in the phosphorylation of STAT5 and STAT3. **(C)** Real-time PCR analysis of IL-17A, RORγt, and Foxp3 in Th17 cells following 48 h of Treg polarization conditions with or without aloperine. The results were from three independent experiments. The values are presented as the mean ± SEM. ∗*p* < 0.05; ∗∗*p* < 0.01; and ∗∗∗*p* < 0.001.

It is well known that Treg is important in the regulation of the local inflammatory environment (Zhang et al., 2016). We showed that the frequencies of Treg were significantly increased in the aloperine treatment group, while the origin for the increased Treg is of great interest to us. Given the fact that Th17 to Treg conversion occurs in animals and humans, we check if aloperine could promote the conversion of Th17 cells into Treg (Obermajer et al., 2014). Interestingly, a significant number of Foxp3/IL-17A double positive cells and Foxp3 single positive cells were observed in the aloperine (plus IL-2 and TGF-β)-treated Th17 cells (Figure 6A). However, the frequencies of IL-17A-positive cells were decreased by aloperine. Moreover, western blot analysis showed that the phosphorylation of STAT5 was significantly increased in the aloperine group, but the level of phosphorylated STAT3 was decreased (Figure 6B). In addition, RT-PCR revealed that the expression of Foxp3 was increased, while IL-17A and RORγt were decreased in the aloperine-treated Th17 cells (Figure 6C). This could explain the lower frequency of Th17 cells and the higher proportion of Treg in the aloperine group during the progressive phase of the disease. In all, these findings indicate that aloperine plays a role in the conversion of Th17 into Treg through tipping the balance of pSTAT5 and pSTAT3.

## Discussion

**FIGURE 7:**
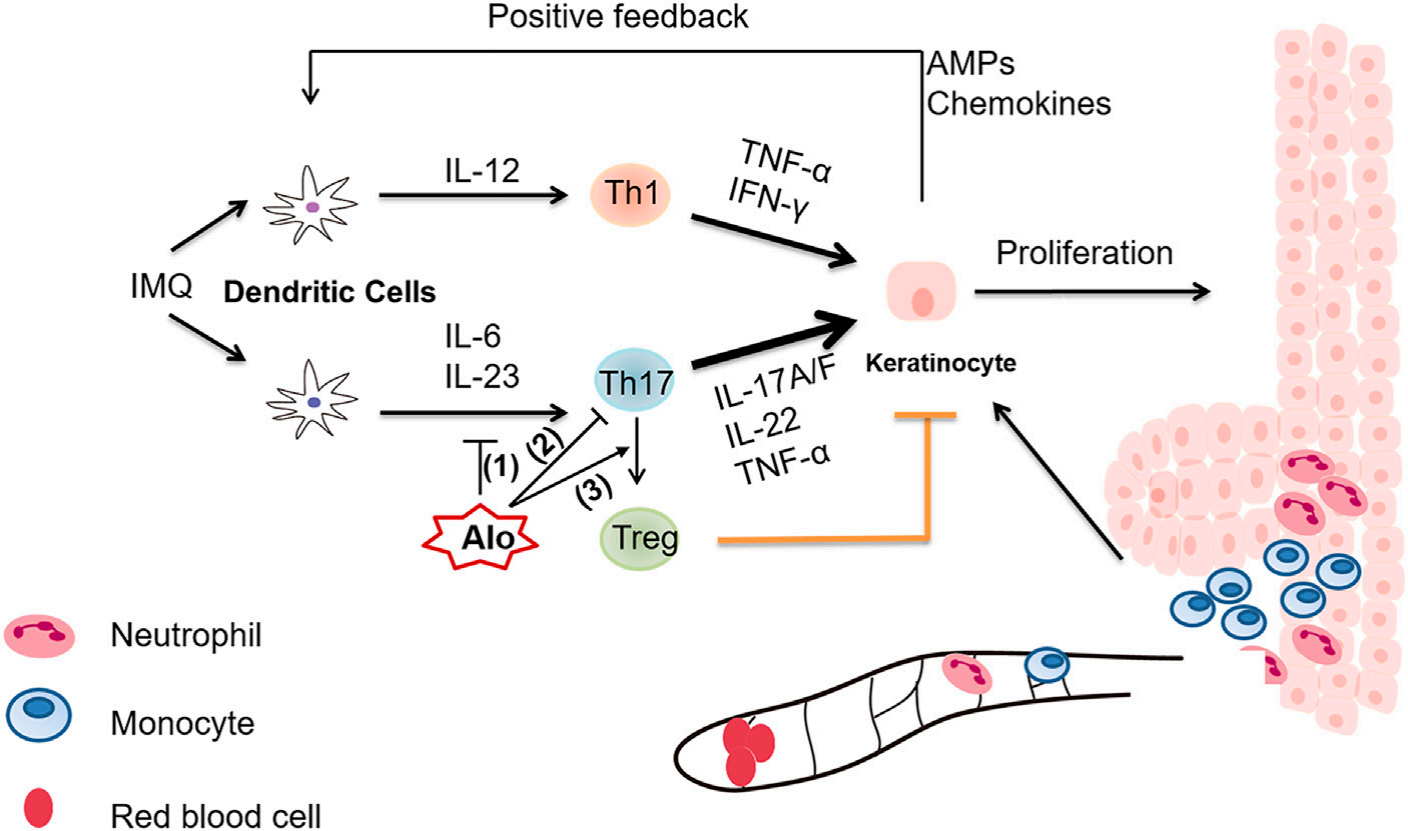
A graphical illustration of aloperine-mediated suppression of the Th17 program in the mouse psoriasis model. (1) Aloperine inhibits the migration, activation, and Th17-inducing capability of DCs. (2) Aloperine directly inhibits Th17 polarization by suppressing the phosphorylation of STAT3. (3) Aloperine promotes the trans-differentiation of Th17 into Treg.

Aloperine is a natural quinolizidine alkaloid extracted from *Sophora alopecuroides L.*, and has been used as an effective therapy in the treatment of inflammatory diseases, such as colitis and ischemia-reperfusion-induced renal injury (Hu et al., 2016). Nevertheless, the detailed mechanism underlying the immune-regulatory role of aloperine is poorly understood. In this study, we sought to investigate the multifaceted effect of aloperine on the Th17 program. Major discoveries in this study are summarized below (Figure 7). First, our data suggested that aloperine exerts suppressive effects on DCs by inhibiting their migration, activation, and cytokine secretion, especially those related to Th17 polarization. Western-blot results indicated that aloperine could inhibit the NF-κB signaling pathway in DC, which is consistent with the previous reports (Ye et al., 2020; Hu et al., 2021). Moreover, as far as we know, we provided the first panorama clarifying the effect of aloperine on the CD4^+^ T-cell differentiation. The results demonstrated that aloperine administration has no effect on CD4^+^IFNγ^+^ Th1 differentiation, and moderately increases the CD4^+^IL-4^+^ Th2 subset, but significantly impairs CD4^+^IL-17A^+^ Th17 differentiation. Mechanistically, aloperine inhibits the phosphorylation of STAT3, which is essential for the polarization of Th17 cells. Previously, we demonstrated that aloperine promotes Treg differentiation *via* suppressing the PI3K/Akt/mTOR signaling pathway. Based on these data, we conclude that aloperine alleviates inflammatory response partially by modulating the CD4^+^ T-cell differentiation. Intriguingly, aloperine directly promotes the trans-differentiation of Th17 into Treg by tipping the balance of pSTAT5 and pSTAT3, which implies a more flexible and subtle effect on the adaptive immune response that aloperine confers. In combination with our previous publication on Treg differentiation (Fu et al., 2017), we conclude that aloperine is efficient to restore the Th17/Treg imbalance, thus serving as a potent therapeutic agent in psoriasis and other immune-related disorders.

Psoriasis is a chronic inflammatory skin disease characterized by skin erythema, plagues, and constant scales derived from excessive proliferation of epithelial cells. IMQ is a TLR7/8 ligand and a potent immune activator of monocytes, macrophages, and DCs, by which it plays a critical role in initiating psoriatic skin inflammation. IMQ-induced psoriasis is established as a rapid and convenient animal model to elucidate the pathogenic mechanism of psoriasis and to screen potential antipsoriasis drugs *in vivo* (Chuang et al., 2018). We therefore explored the therapeutic effect on psoriasis and the related mechanisms in an IMQ-induced psoriasis model. Previous studies demonstrated strong evidence for the role of the IL-23/Th17 axis both in the psoriasis patients and animal models. IL-23, mostly derived from the activated DCs, plays an important role in the proliferation and maintenance of immune response. Intradermal injection of IL-23 in mice led to apparent skin inflammation with histopathological features resembling psoriasis (Lindroos et al., 2011; Rizzo et al., 2011). In the present study, we found that aloperine not only impaired Th17 differentiation directly but also inhibited the activation of dendritic cells, thereby reducing the expression and secretion of proinflammatory cytokines, especially IL-23 and IL-1β. Thus, our data support that aloperine ameliorates IMQ-induced psoriatic skin injury by creating a Th17-disfavoring microenvironment.

Evidence also suggests that neutrophils, monocytes, and monocyte-derived DCs were accumulated in the dermis during the early phase, and macrophages transiently increased in the epidermis and dermis during the late phase (Lee et al., 2018). These findings highlight their key role in inducing the psoriasis-like skin disease. In our study, we observed that aloperine had no effect on the infiltration of neutrophils but could inhibit the migration of DCs and macrophages through the application of an air pouch model. Our finding emphasized the pivotal functions of antigen-presenting cells (DCs in particular) during the early phase of psoriasis development, which could be a perfect time window for the intervention by either aloperine or other chemical compounds.

Th17/Treg imbalance is known to trigger and accelerate the progression of psoriasis (Shi et al., 2019). In psoriatic patients, the impaired function of Treg cells is mediated by the phosphorylation of STAT3 (Yang et al., 2016; Zhang et al., 2016). Treg cells from psoriatic patients could reprogram into IL-17A-producing Th17 cells, which are identified in the peripheral blood and skin lesions, suggesting that the stability of Treg is compromised in the psoriatic inflammation environment (Singh et al., 2013). Our group previously found that aloperine promoted Treg differentiation and activation in mice by inhibiting the PI3K/Akt/mTOR signaling pathway. In this study, we found that the proportion of Treg was increased in the aloperine treatment group, which is consistent with the previous study. We further checked whether increased Treg in the aloperine treatment group was derived from the conversion of Th17 cells. Excitingly, we found that aloperine could alter the pSTAT3/pSTAT5 ratio and promote the conversion from Th17 to Treg cells. These findings suggested that aloperine could improve the skin local immune environment through affecting Th17/Treg balance. Nonetheless, the limitation of this study is that we did not fully identify the specific molecules that interact with aloperine directly, which would be the focus of later investigations.

In summary, topical administration of aloperine ameliorates IMQ-induced psoriasis *via* its versatile suppressive effect on the Th17 program. These findings provide important mechanistic insights into the therapeutic benefits of aloperine, which holds the potential as a candidate drug for the treatment of various inflammatory diseases.

## Data Availability

The datasets presented in this study can be found in online repositories. The names of the repository/repositories and accession number(s) can be found in the article/Supplementary Material.
