# Supplementary material for: Aloperine Ameliorates IMQ-Induced Psoriasis by Attenuating Th17 Differentiation and Facilitating Their Conversion to Treg
Source: Front Pharmacol. 2022 Jun 1;13:778755. doi: 10.3389/fphar.2022.778755 (PMC9198605; doi:10.3389/fphar.2022.778755)
Supplement: Supplementary file 1 [file Presentation1.PPTX]

## Slide 1
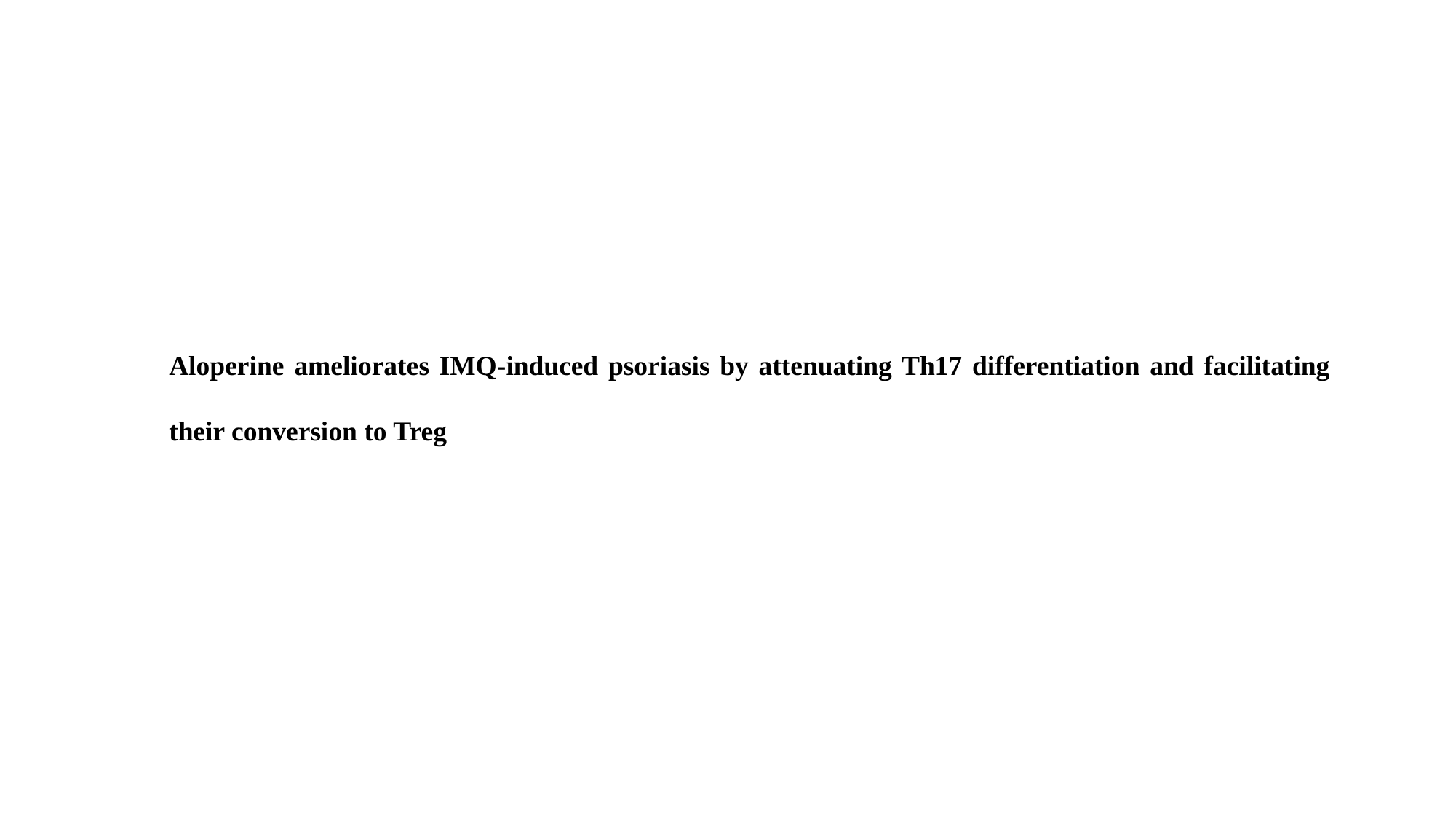

Aloperine ameliorates IMQ-induced psoriasis by attenuating Th17 differentiation and facilitating their conversion to Treg

## Slide 2
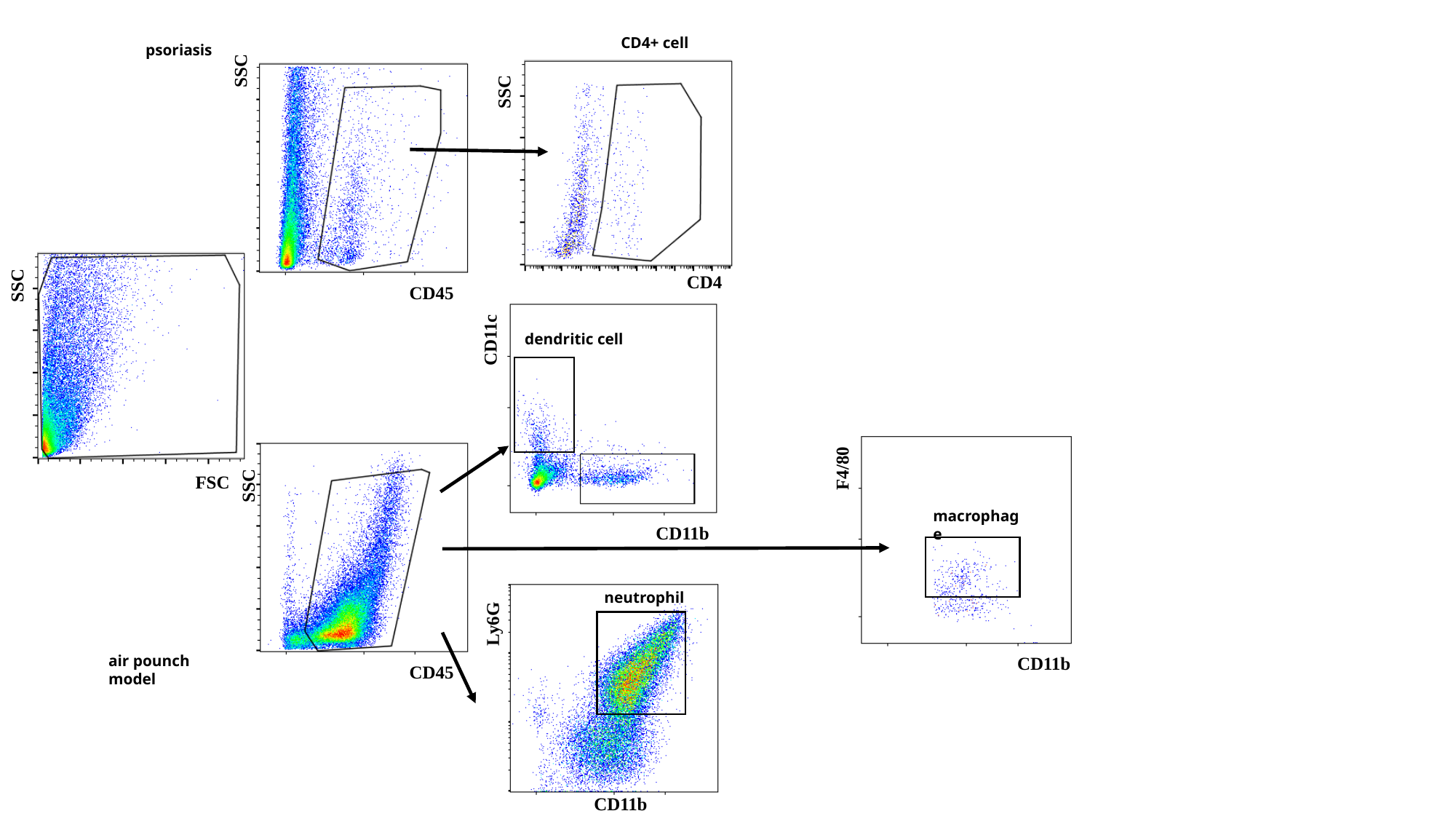

CD4+ cell
psoriasis
SSC
SSC
SSC
CD4
CD45
CD11c
dendritic cell
F4/80
FSC
SSC
macrophage
CD11b
neutrophil
Ly6G
air pounch model
CD11b
CD45
CD11b
