# Supplementary material for: Aloperine Ameliorates IMQ-Induced Psoriasis by Attenuating Th17 Differentiation and Facilitating Their Conversion to Treg
Source: Front Pharmacol. 2022 Jun 1;13:778755. doi: 10.3389/fphar.2022.778755 (PMC9198605; doi:10.3389/fphar.2022.778755)
Supplement: Supplementary file 2 [file DataSheet1.PDF]

Aloperine ameliorates IMQ-induced psoriasis by attenuating Th17 differentiation and facilitating their conversion to Treg

Western-blot original data

Figure 3F

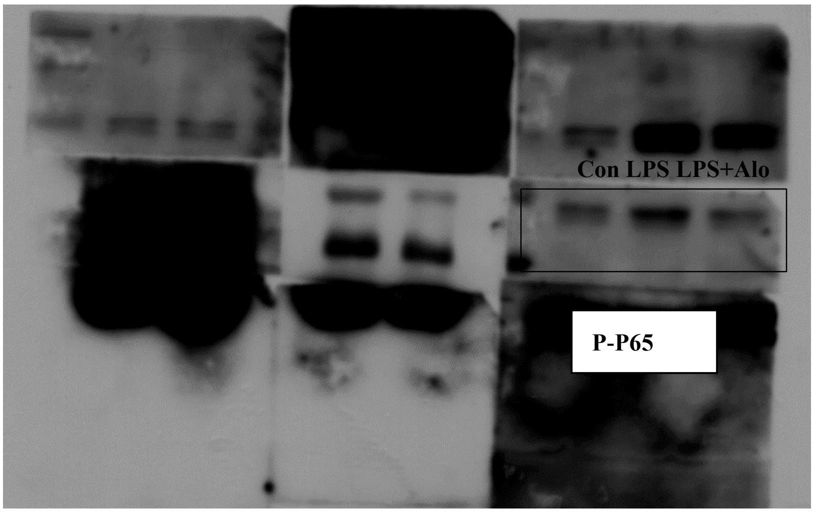

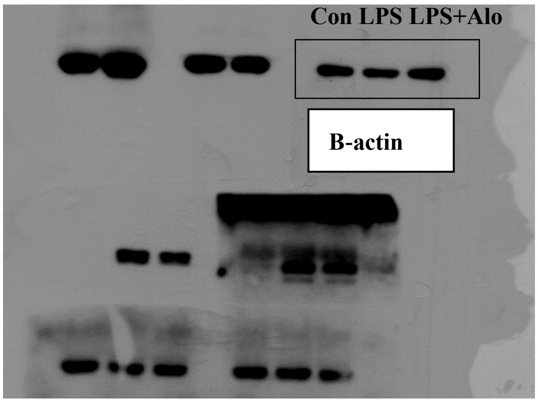

Figure 5D

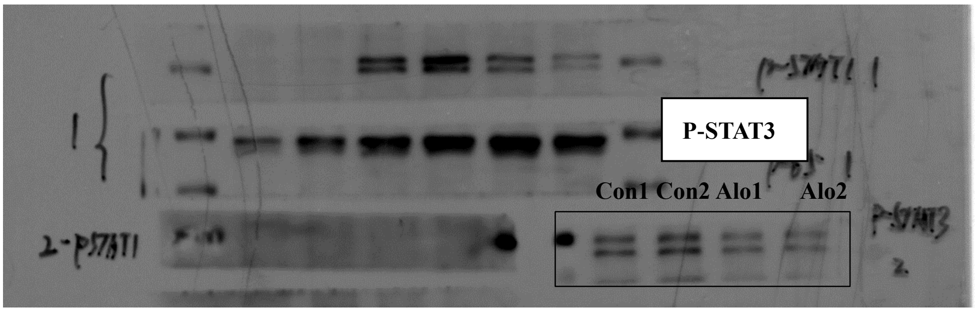
P-STAT3
Con1 Con2 Alo1 Alo2

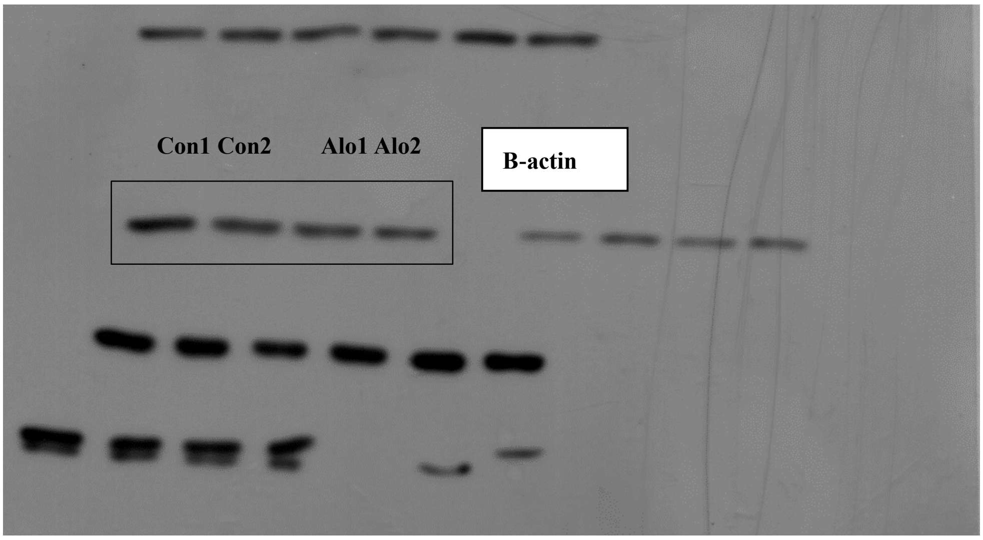
Con1 Con2 Alo1 Alo2
B-actin

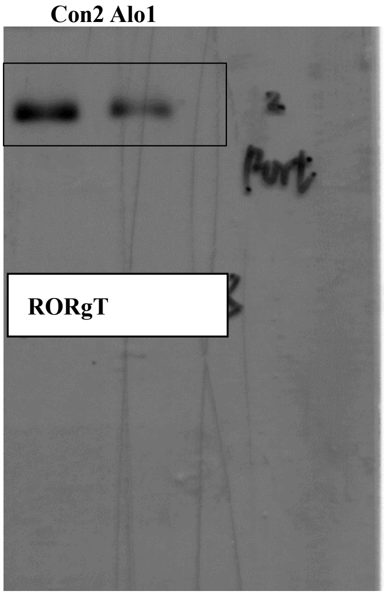
Con2 Alo1
RORgT

Figure 6B

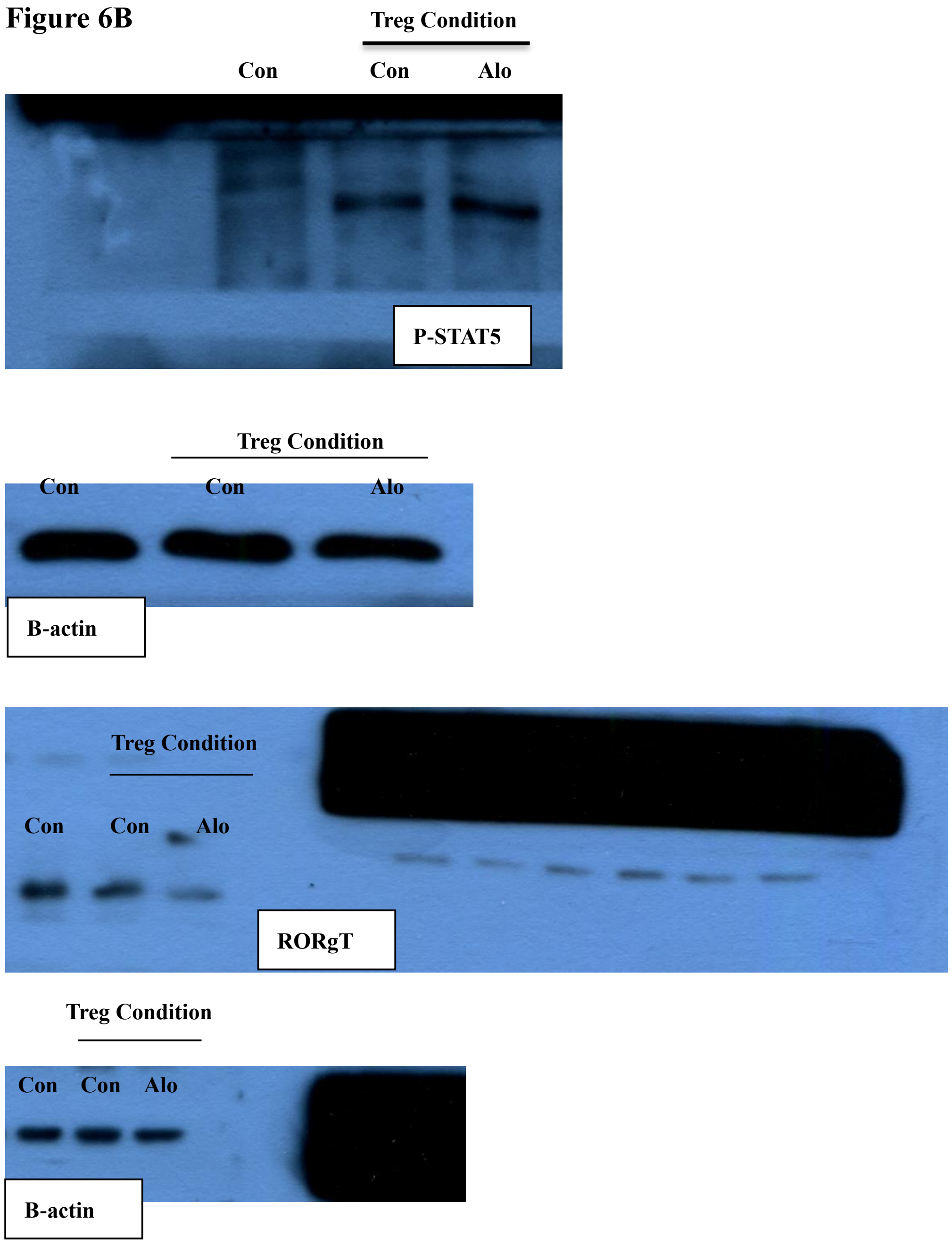
Treg Condition
Con
Con
Alo
P-STAT5
Treg Condition
Con
Con
Alo
B-actin
Treg Condition
Con
Con
Alo
RORgT
Treg Condition
Con
Con
Alo
B-actin
